# Supplementary material for: A pan-cancer analysis of homeobox family: expression characteristics and latent significance in prognosis and immune microenvironment
Source: Front Oncol. 2025 Feb 6;15:1521652. doi: 10.3389/fonc.2025.1521652 (PMC11840236; doi:10.3389/fonc.2025.1521652)
Supplement: Supplementary Figure 1 — Mutations of each HOX gene in 32 cancers (TCGA, PanCancer Atlas) by cBioportal. [file DataSheet1.zip › Suppl.files/raw code.zip!/code/4.survival/survivalplot.zip!/survivalplot/HOXD8_OS.LGG.pdf]

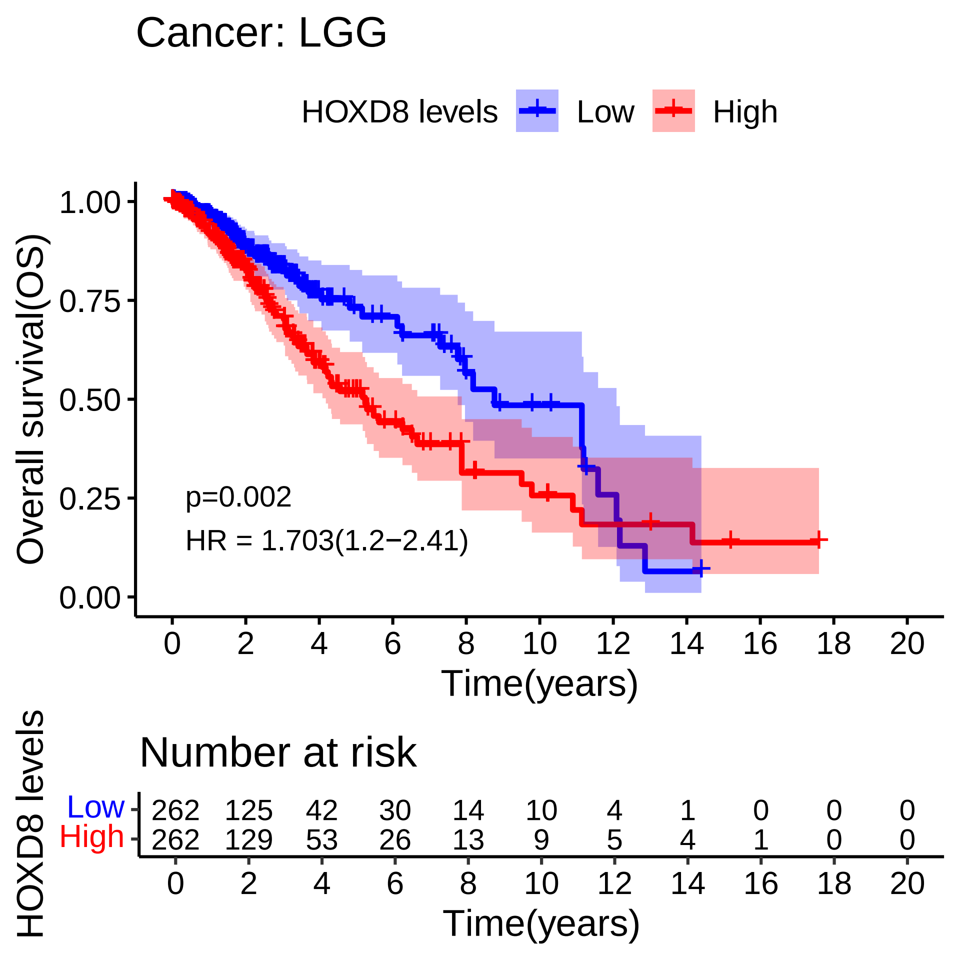

Cancer: LGG
HOXD8 levels
Low
High
Overall survival(OS)
1.00
0.75
0.50
0.25
0.00
p=0.002
HR = 1.703(1.2−2.41)
0 2 4 6 8 10 12 14 16 18 20
Time(years)
Number at risk
HOXD8 levels
Low 262 125 42 30 14 10 4 1 0 0 0
High 262 129 53 26 13 9 5 4 1 0 0
0 2 4 6 8 10 12 14 16 18 20
Time(years)
